# Monitoring, Mapping, and Modeling Spatial–Temporal Patterns of PM_2.5_ for Improved Understanding of Air Pollution Dynamics Using Portable Sensing Technologies

**DOI:** 10.3390/ijerph17144914

**Published:** 2020-07-08

**Authors:** Ronan Hart, Lu Liang, Pinliang Dong

**Affiliations:** Department of Geography and the Environment, University of North Texas, 1155 Union Circle, Denton, TX 76203, USA; ronanhart92@gmail.com (R.H.); pinliang.dong@unt.edu (P.D.)

**Keywords:** particulate pollution, landscape pattern, mobile monitoring, GIS, Lidar

## Abstract

Fine particulate matter with an aerodynamic diameter of less than 2.5 µm (PM_2.5_) is highly variable in space and time. In this study, the dynamics of PM_2.5_ concentrations were mapped at high spatio-temporal resolutions using bicycle-based, mobile measures on a university campus. Significant diurnal and daily variations were revealed over the two-week survey, with the PM_2.5_ concentration peaking during the evening rush hours. A range of predictor variables that have been proven useful in estimating the pollution level was derived from Geographic Information System, high-resolution airborne images, and Light Detection and Ranging (LiDAR) datasets. Considering the complex interplay among landscape, wind, and air pollution, variables influencing the PM_2.5_ dynamics were quantified under a new wind wedge-based system that incorporates wind effects. Panel data analysis models identified eight natural and built environment variables as the most significant determinants of local-scale air quality (including four meteorological factors, distance to major roads, vegetation footprint, and building and vegetation height). The higher significance level of variables calculated using the wind wedge system as compared to the conventional circular buffer highlights the importance of incorporating the relative position of emission sources and receptors in modeling.

## 1. Introduction

Fine particulate matter with an aerodynamic diameter of less than 2.5 µm (PM_2.5_) is a mixture of extremely small particles with adverse consequences for human health and the environment [1,2,3]. Urban air pollution has surfaced as a significant global environmental health concern because 55% of the world’s population lives in urban areas [4], and more than 80% of urban dwellers live in areas with the air quality levels exceeding World Health Organization guideline limits [5].

The spatio-temporal patterns of PM_2.5_ are highly variable within cities [6,7], because the formation and dispersion of PM_2.5_ involve complex physical and chemical processes resulting from human–atmosphere–biosphere interactions [8], and urban ecosystems manifest the highest degree of heterogeneity and uncertainty in these interactions [9]. For example, PM_2.5_ is highly concentrated along and near arterial roads [10], and particle pollutants can vary five to eightfold within an individual city block [7,11]. In turn, increasing empirical evidence of localized pollution patterns has led to a growing interest in assessing air pollution at the intra-urban scale in many disciplines [12,13,14,15,16,17,18].

Although all major U.S. cities have monitoring stations that provide continuous measurements of criteria air pollutants, the number of these stations is usually too low to capture small-scale spatial variability in the pollutants’ distribution [19]. For instance, the Dallas–Fort Worth Metroplex (DFW), home to approximately 7 million people, has only six stations monitoring PM_2.5_, and most are located in old city areas. The expanded use of low-cost air quality sensors is recommended by the Environmental Protection Agency (EPA) as the “new paradigm for air quality monitoring” [20]. Unlike traditional stationary measurements that require high operational cost and expertise, low-cost sensors are easy to use, portable, and affordable. They offer additional avenues to refine the spatio-temporal characterization of air quality by allowing direct citizen engagement and wide community deployment.

The concentration of particle pollutants at a specific location and time is determined by many factors, including emission sources, meteorology, landscape features, and physicochemical transformations. To understand the driving factors of the spatio-temporal dynamics, it is common to statistically link critical environmental factors—especially those that influence pollutant emission intensity and dispersion efficiency—to site measurements. However, some overlooked facets include the quantity and quality of input air pollution data, the 3D representation of predictor variables, and the wind effects. For instance, to properly specify a regression model, a minimum of 40–80 sampling locations is suggested [21]. However, most previous models were built upon a small number of fixed-site measurements [22]. Moreover, most traditionally used explanatory variables only describe the horizontal landscape, such as green space distributions [23]. Rarely do those variables account for the dispersion environment (e.g., street canyons, position and dimensions of buildings, and road width). Finally, the explanatory variables are typically calculated using point estimates at a specific location, or based on circular [22] or varying-distance buffers [24]. Those buffers apply equal weights to emission sources around a receptor, irrespective of the prevailing meteorological conditions and the relative positions of sources and receptors [25]. This limitation could be especially exaggerated when the model is developed at fine resolution.

The goal of this study is to characterize the intra-urban PM_2.5_ variability at fine resolution and determine significant contributors via an integrated “monitoring–mapping–modeling” approach, by developing new models and variables to overcome the aforementioned challenges. We utilized the mobile monitoring data to reflect real-time fluctuations in local air quality and thus enrich the model input data. New variables, such as building and vegetation heights, will be needed for an enhanced 3D representation of land use and the physical characteristics of dispersion environments [26]. Moreover, to account for the wind effects, a new buffer system that can incorporate the relative position of emission source and receptor was deployed.

## 2. Study Area

**Figure 1:**
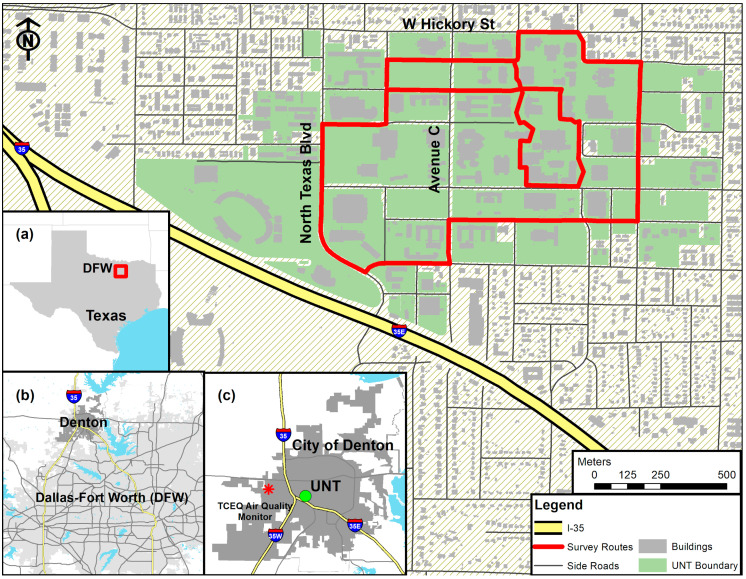
The survey route map. Three inserts separately display: (**a**) location of the Dallas–Fort Worth Metroplex (DFW) in the State of Texas, United States; (**b**) location of the city of Denton in DFW; (**c**) location of University of North Texas (UNT) and the Texas Commission on Environmental Quality (TCEQ) Air Quality Monitor in the City of Denton.

This study was conducted on the University of North Texas (UNT) campus, which is located in Denton County, Texas—the northern part of the Dallas–Fort Worth metroplex (hereafter referred to as DFW; officially known as the ‘Dallas–Fort Worth–Arlington metro area’ by the U.S. Census Bureau; Figure 1a). The DFW is ranked as the fourth most populous metropolitan area in the U.S. and has the largest population growth from 2017–2018 with a gain of 131,767 (1.8%) residents in the nation [27]. The city of Denton is located at the northbound of DFW (Figure 1b), where the major Interstate 35 (I-35) rejoins from I-35E and I-35W. Given its importance as a transportation node in DFW, Denton has experienced extensive urbanization with an increase of 18% in population since 2010 [27]. UNT is the largest higher education sector in Denton, with 38,087 enrolled students and 3671 full-time employees in 2018 [28].

Air quality in Denton has been consistently ranked among the worst in Texas. North Texas asthma rates are reported as three times higher than the state’s average and twice as high as the nation’s average [29]. Emissions from idling trucks, construction, hydraulic fracturing, and the brunt of pollution blown from factory plants from South Dallas are all known to be major contributing factors to Denton’s poor air quality [30]. However, within the total area of 2470 km^2^ in Denton, there is only one Texas Commission on Environmental Quality (TCEQ) monitoring station that measures PM_2.5_ (Figure 1c). This station is located at the TCEQ Denton Airport South location, approximately 4 km west of UNT and 6.5 km from the city of Denton’s urban center, where PM_2.5_ levels would be expected to be representative for the whole city. 

Given the magnitude of the population impacted, relatively poor air quality, and lack of monitoring sites, UNT is a unique place to conduct mobile air quality monitoring research. Compared to K-12 school settings, very few studies have investigated exposure to particulate matter in the university setting, and previous studies largely focused on environmental quality factors and perception-based measures [31,32]. Thus, there is a critical need to characterize and understand air quality on campus.

## 3. Methodology

### 3.1. Mobile Air Monitoring Surveys

In this study, mobile air monitoring surveys were conducted on UNT’s main campus using a bicycle-based sampling system along a fixed route. Details of the mobile devices, selection of survey routes, and methods of raw data aggregation are specified below.

#### 3.1.1. Device

**Figure 2:**
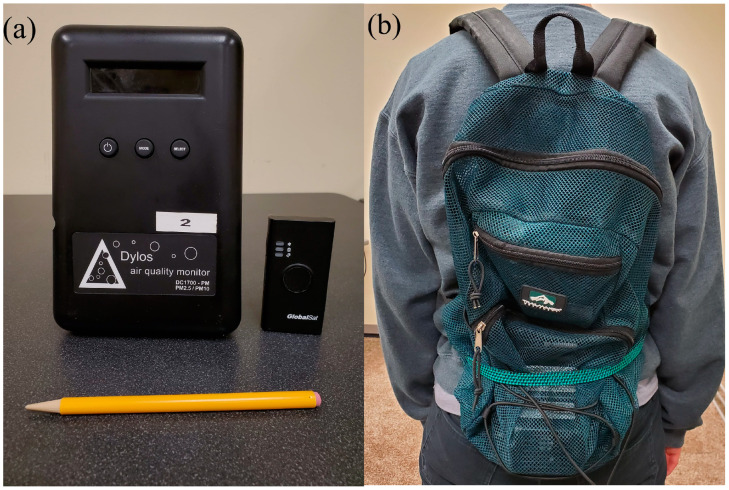
(**a**) The Dylos sensor and GPS units used to measure outdoor fine particulate matter with an aerodynamic diameter of less than 2.5 µm (PM_2.5_) concentration and record travel trajectory, respectively, with a pencil for size reference; (**b**) the outfit of the two devices in a meshed backpack.

Bicyclists were outfitted with a Dylos DC1700 particle counter (Dylos Corporation, Riverside, California) and a GlobalSat DG-500 GPS unit (GlobalSat WorldCom Corp., New Taipei City, Taiwan) strapped inside of a mesh backpack that allows free airflow (Figure 2). The Dylos 1700 model is a battery-operated, laser particle counter that measures the particle concentrations for PM_2.5_ and PM_10_ (µg/m^3^) for each minute. Here, only PM_2.5_ concentrations were used. The GPS unit collects positional information every 5 s.

#### 3.1.2. Survey Routes

Our mobile survey route contains two paths to span different levels of traffic and types of land use. One external path follows the major vehicle and bus traffic on campus to capture the pollution distribution patterns resulted from emissions. One internal path uses the paved pathways solely for pedestrians and bicyclists in the interior of campus in order to show contrast. The route is 5.35 km in total and takes an average of 30 min to bike through. Mobile monitoring was conducted every four hours repeatedly during the workdays of 28 January 2019, to 8 February 2019: 07:00, 11:00, 15:00, and 19:00. The hours of 07:00. and 15:00 are campus rush hours when people are entering and leaving campus; 11:00 is the class passing time and during lunch break when people are often out and walking, so the likelihood of personal exposure to pollutants could be the highest; 19:00 is the evening rush hour on I-35 and traffic on campus is mainly from students taking evening classes. In total, 34 sampling runs were completed. The raw data and aggregated survey data were provided in Files S1 and S2.

#### 3.1.3. Data Aggregation 

For visualization and data polishing purposes, we aggregated the point-based mobile measures at equal interval segments along the sampling routes.

The sampling frequencies of PM_2.5_ and GPS data were 1 min and 5 s, respectively. The PM_2.5_ concentration data were joined with the GPS data if the 5 s time stamp of GPS data fell within the 1 min timestamp of the PM_2.5_ data. Then, we aggregated measurements at a 50 m distance along the sampling routes. This resolution is selected as it is fine enough to reveal spatial variability while ensuring a sufficient number of measurements per segment (approximately 4 points per segment). Furthermore, the dataset of instantaneous measurements were converted into estimates of median concentration for individual 50 m segment via data reduction algorithms. First, each 5 s measurement was snapped to the nearest segment on the basis of its corresponding GPS coordinates. Second, for each segment at each sampling run, we computed the median of all the snapped measurements. The median concentration was chosen as the core estimate of central tendency for the segment over the mean considering the potentially small number of extreme outlier observations. The standard deviation was also calculated for each segment as a representation of the within-segment PM_2.5_ variability. The raw data and aggregated survey data are provided in Files S1 and S2.

### 3.2. Development of Explanatory Variables

A range of predictor variables that have been proven useful in estimating the pollution level was derived from GIS datasets. Sixteen variables that fell into three categories, separately weather conditions, proximity to emission sources, and urban morphology were developed (Table 1).

#### 3.2.1. Meteorological Variables

Meteorological conditions, including wind, temperature, precipitation, and relative humidity, can affect PM_2.5_ concentrations [33,34]. Each of those variables has been well documented to have effects on air pollutants, but the relationships remain uncertain.

(1) Temperature affects chemical reactions and atmospheric turbulence that determine the formation and diffusion of particles [35,36]. Some studies indicate that higher temperature promotes the photochemical reaction between PM_2.5_-forming precursors and thus elevates particle mass [36]. Other studies reveal that when the temperature rises, thermally induced air convection becomes frequent, which leads to the diffusion and dilution of particulate matter [37,38].
(2) Humidity is closely related to the pollutant level. PM_2.5_ concentrations tend to increase while humidity is low. Once the humidity reaches a high value, particles will absorb moisture and condense, which leads to dry deposition of the particles to the ground and thus results in a lower concentration of PM_2.5_ in the air [39]. Here, we used the dew point as a measure of atmospheric moisture. A higher dew point indicates more moisture in the air.
(3) Wind speed, direction, and gust are crucial indicators of atmospheric activity. They greatly affect air pollutant transport and dispersion [40]. Wind speed affects the pollutant concentration [41], and wind direction determines where the pollutant blows from and disperses to [42,43]. Wind gust is the rapid fluctuations in the wind speed with a variation of 10 knots or more between peaks and lulls, and it indicates the maximum instantaneous wind speed. Many previous research methods use long-term average wind speed and direction to estimate the pollution concentrations [44,45]. However, wind can fluctuate rapidly over the short term and its influence on the pollutant dispersion and deposition processes is in a timely manner. Thus, the dependencies between wind and PM_2.5_ concentrations are multidirectional and time-sensitive [46]. In this study, we designed a wind wedge system to account for the real-time changes of wind, which will be elaborated in Section 3.3.

We collected data for all meteorological variables (temperature, dew points, wind direction, wind speed, and wind gust) from the National Oceanic and Atmospheric Administration [47]. Meteorological data were reported hourly and were joined to the corresponding hour of the survey data. We joined data from surveys that spanned two hours (e.g., 10:50 to 11:20) to two sets of meteorological data based on the time stamp.

#### 3.2.2. Proximity to Emission Sources

The near-road PM_2.5_ increment has been proven statistically significant in many studies [48,49] and the magnitude of increment can range from 10–23% as compared to background concentration [50,51]. UNT has many roadways with high traffic volume within and surrounding its campus, notably North Texas Blvd, W Hickory St, Avenue C, and I-35E (Figure 1). We acquired the road network data from Denton County GIS office. Considering the differences between major and minor roads in traffic density, these two categories of roads were applied separately in further analysis.

#### 3.2.3. Urban Morphology: Airborne Image-Derived Horizontal Landscape Pattern

**Figure 3:**
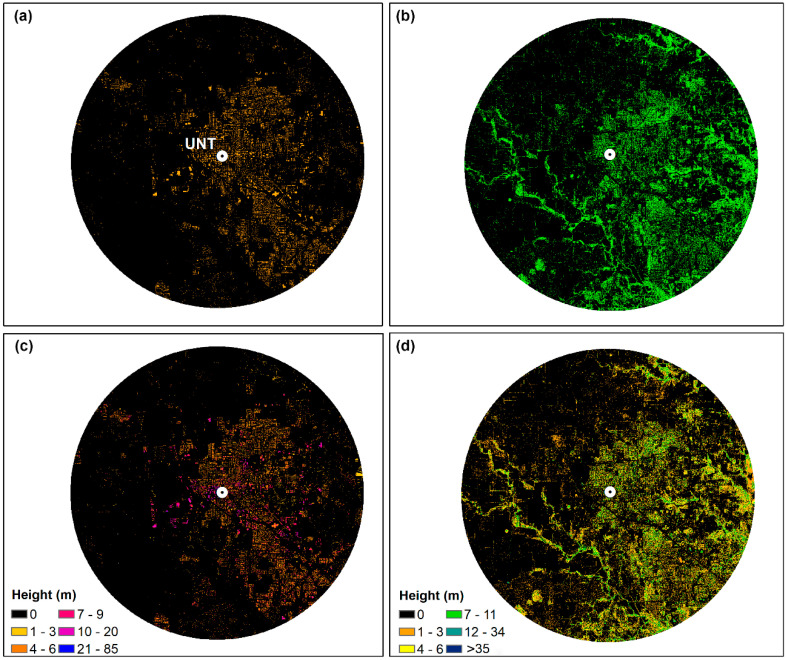
Maps of (**a**) building footprint, (**b**) vegetation footprint, (**c**) building height, and (**d**) vegetation height in meters in a 6-mile radius from the study area.

To describe horizontal landscape patterns at a fine spatial resolution, we generated a land-cover map based on the United States Department of Agriculture (USDA) National Agricultural Imagery Program (NAIP) aerial photographic imagery. NAIP color-infrared imagery consists of four 8-bit spectral bands (red, green, blue, and near-infrared) at 1 m spatial resolution. The latest acquisition year for our study area is 2016. To better distinguish between vegetation and non-vegetation, we also generated a vegetation indices layer of Normalized Difference Vegetation Index (NDVI), which is a quantitative index of greenness, using the red and near-infrared bands of NAIP [52]. Aided with 240 visually interpreted training samples, we applied the supervised classification to the combined four raw spectral bands and one NDVI layer. The final map reached an overall accuracy of 86% by validating against an independent set of test samples. Finally, the footprints of the buildings and green space were derived to quantify the urban morphology at the horizontal plane (Figure 3a,b). Adequate evidence has suggested that vegetation and built-up were significant landscape elements in determining air quality [53]. Urban green space can provide cleaner air, especially in its vicinity areas [46,54]. The density of urban built-up was found to be positively correlated with air pollution distribution [55]. The full description of methodology and the results can be found in File S3.

#### 3.2.4. LiDAR-Derived 3D Representation of the Built Environment and Trees

**Figure 4:**
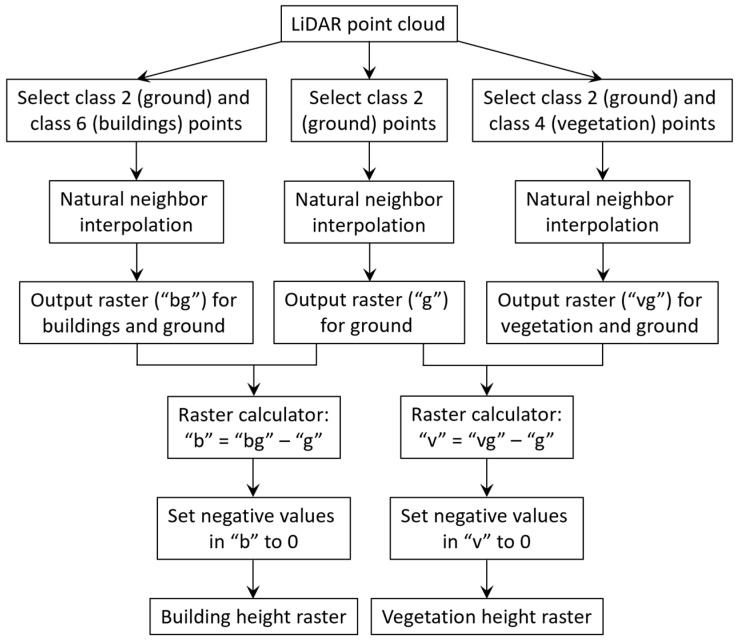
Flowchart for Light Detection and Ranging (LiDAR) data processing.

We used airborne Light Detection and Ranging (LiDAR) data to generate rasters for building and vegetation height. The airborne LiDAR data in the study area were acquired in March 2011 with an average point density of 4.8 points/m^2^ and classified into seven classes: 1—unclassified, 2—ground, 4—vegetation, 6—buildings, 7—low point (noise), 9—water, and 13—bridges and culverts. To reduce errors caused by spatial interpolation, several steps were used to create a building height raster and a vegetation height raster, with a cell size of 1 m by 1 m. The steps are shown in the following flowchart (Figure 4). The derived building height raster and vegetation height raster are shown in Figure 3c,d.

### 3.3. Wind Wedge-Based Explanatory Variable Calculation

**Figure 5:**
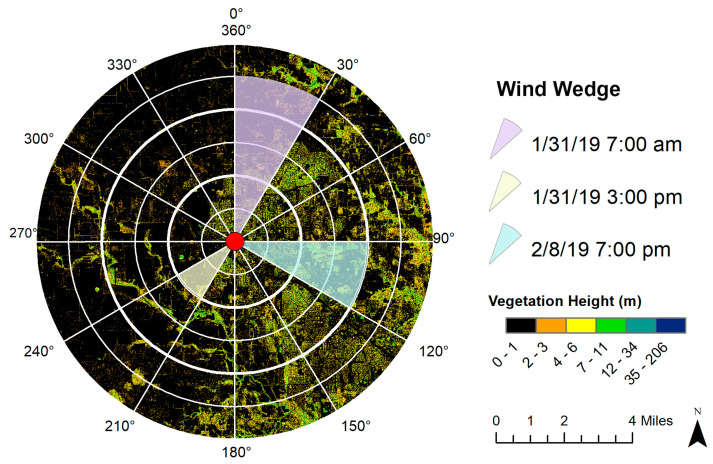
Wind wedge used in the explanatory variable calculation. Dates and the associated wind parameters are only for illustration purposes.

As stated in Section 3.2.1, wind direction and speed are key parameters in estimating the landscape–air pollution relationship. As wind conditions may vary substantially, the spatial predictors need to be calculated by incorporating wind effects. We first discretized the 360° wind field into a set of 12 30° wind wedges (Figure 5). The direction of the wedge depends on the wind direction, and the radius of the wedge relies on the wind speed. To determine the radius, we used the Beaufort Wind Scale [56]. For example, Figure 5 illustrated the wind wedge overlaid on top of vegetation height layer. The three wedges corresponded to three wind scenarios. If the wind speed was 17 miles per hour and the wind direction was 15° from north, then the wedge would look similar to the purple wedge in Figure 5; or if the wind speed was 5 miles per hour and the wind direction was 215° from north, then the wedge would look similar to the yellow wedge in Figure 5. The average of vegetation and building heights and summation of vegetation and building footprints were calculated within the wedge using the focal statistics tool and used in further analysis.

To provide a more holistic assessment of the wind wedge system, we also calculated the average and sum of the four spatial predictors based on the traditional circular buffer with a variable-distance radius. The buffer radius was set to be the same as that of the wedges to include the influence of the surrounding urban landscape but with no consideration of wind direction.

### 3.4. Panel Data Analysis

We employed the panel data analysis to identify the most important variables in explaining the PM_2.5_ spatio-temporal patterns. The panel data analysis is a quantitative analytical method that deals with observations from multiple locations over multiple periods. Its capacity in analyzing the characteristics and changes from both the cross-section and time-series dimension of data surpasses conventional models that purely focus on one dimension [57]. The estimation equation for the panel data model in this study is given as:

PM_2.5it_ = β_0_ + β_1_ × WDIR + β_2_ × WSP + β_3_ × GST + β_4_ × T + β_5_ × H + β_6_ × Dis_major_ + β_7_ × Dis_major_ + β_8_ × Dis_bus_ + β_9_ × VegFP_wedge_ + β_10_ BuildFP_wedge_ + β_11_ × VegHt_wedge_ + β_12_ × BuildHt_wedge_+ β_13_ × VegFP_buffer_ + β_14_ × BuildFP_buffer_ + β_15_ × VegHt_buffer_ + β_16_ × BuildHt_buffer_+ ℇ_it_

where the subscripts i and t refer to route segment and sampling time, respectively. β_0_ is the intercept parameter, β_1_ to β_16_ are the estimates of slope coefficients, and ε is a random error. All explanatory variables are transformed into natural logarithms for data normalization. All variables are calculated in the natural logarithm.

There are two methods in panel data analysis to obtain estimates: separately fixed effects estimator and random effects estimator. Depending on the form of the error term, the fixed effects estimator assumes that each subject has its specific characteristics due to inherent individual characteristic effects in the error term, thereby allowing differences to be intercepted between subjects. The random effects estimator assumes that the individual characteristic effect changes stochastically, and the differences in subjects are not fixed in time and are independent between subjects. To identify the right estimator, the Hausman specification test was used to choose between models [58], with the null hypothesis that random effects model yields consistent and efficient estimates [59]. If the null hypothesis is rejected, the fixed effects model will be selected for further inferences and vice versa.

## 4. Results and Discussion

### 4.1. Diurnal and Daily Variation of PM_2.5_ Concentration

**Figure 6:**
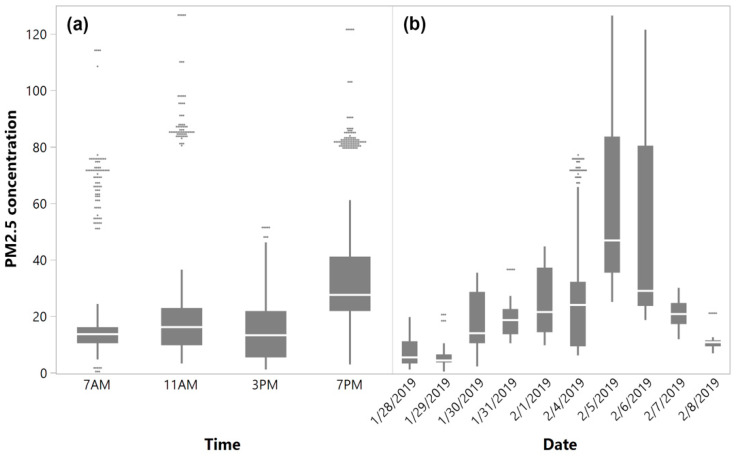
Box plots showing (**a**) the diurnal variation and (**b**) daily variation of PM_2.5_ concentrations (µg/m^3^).

The mobile monitoring collected PM_2.5_ concentrations vary diurnally and daily on the UNT campus over the sampling period. The diurnal profiles of PM_2.5_ concentration are shown within box plots for each sampling hour in Figure 6a. For diurnal variation, the average concentration started low at 07:00 and slightly increased at 11:00. The concentration reached its lowest point at 15:00 and peak around 19:00. However, the differences between 07:00, 11:00, and 15:00 concentrations were very subtle. Lower values in the PM_2.5_ prior to the morning rush hours can largely be attributed to the less accumulated soot particles emitted from vehicular sources. Additionally, the atmospheric conditions are relatively stable around 07:00, and thus dispersion is limited. As the day progresses, increased solar radiation promotes photochemical reactions that lead to the formation of secondary aerosols [60] and contribute to overall increased PM_2.5_ mass. While during the late evening rush hours, PM_2.5_ reached its highest level. This similar pattern has also been observed in several different places, such as Guangzhou, China [61] and a location near downtown Los Angeles, USA [62], where a freeway is also nearby. It was reasoned that the resuspension of local road dust induced by a higher speed of traffic and heavy-duty vehicle, coupled with a lower mixing height during the winter might be responsible for the increase in the PM mass. The UNT campus is also in very close proximity (approximately 200 m) to I-35, and the higher traffic volume on local roads due to evening classes can be another reason for this increase.

The variation of daily-averaged PM_2.5_ concentration is shown within box plots for each sampling day in Figure 6b. For the first week of sampling (28 January 2019–1 February 2019), the mean daily concentration gradually increased from Monday to Friday, except for Tuesday. The second week of sampling (4–8 February 2019) shows a completely different temporal pattern, with Tuesday as the peak day and followed by a gradual decrease until Friday. It is not surprising to observe such a wide range of variations on a weekly basis. The PM_2.5_ concentration is largely determined by the PM chemical composition, which can be affected by multiple factors including emission sources, thermal conditions, and atmospheric mixing and dilution by vertical convection [62]. As those factors change dynamically, the daily averaged PM_2.5_ concentration would vary accordingly.

### 4.2. Spatial Characterization of Intra-Urban PM_2.5_ Gradient

**Figure 7:**
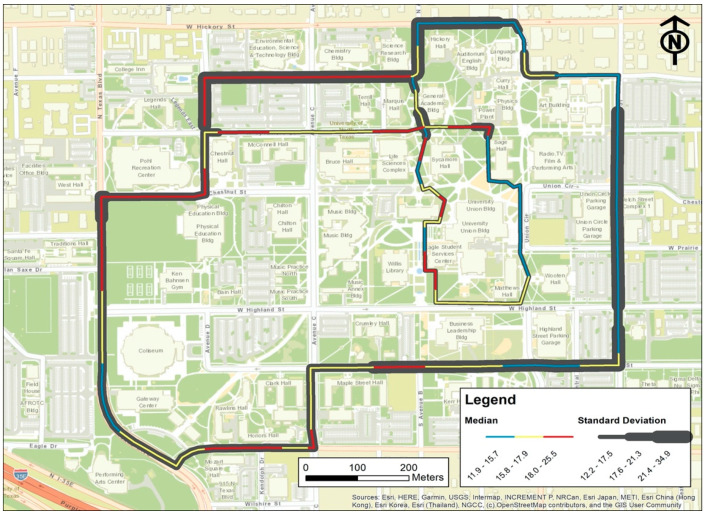
The average and standard deviation of PM_2.5_ concentration (µg/m^3^) during the survey period for each route segment. Colors indicate the mean, and the gray buffer around the lines indicate the range of standard deviation.

We visualized the median PM_2.5_ concentration value of each segment to display the spatial pattern of campus PM_2.5_ at a scale of 50 m (Figure 7). The median values were categorized into three levels: low, medium, and high. Accordingly, each segment is displayed in the color gradient from blue to red. The individual segment was also encompassed by a gray-shaded buffer, of which thickness represents the degree of the standard deviation from the pooled data for each segment.

Considerable PM_2.5_ variation was found within routes. Evidently, the external path, where vehicle and bus traffic is allowed, has all the segments with the heaviest PM_2.5_ pollution (the red category). Areas with good air quality exist in the internal path, which is designated only for pedestrians and bicyclists. The majority of segments in the internal zone are in the lowest category, and only two segments are in the medium category. The variation of the measured PM_2.5_ is also larger for the external path than the internal path. For the inner core of campus, because the pollution from the major traffic roads decays dramatically over 100 m distance [63], much reduced and even no pollution from vehicles should be anticipated. Moreover, more open space and natural environment in the internal path can accelerate the airflow and collectively lead to lower PM_2.5_ values.

### 4.3. Determinants of PM_2.5_ Spatio-Temporal Variation

Two panel data analysis models were applied to determine the major PM_2.5_ pollution contributors on the UNT campus. The Hausman specification test identified the fixed effects model as the better estimator, and hereafter, only the results from the fixed effects model were used for discussion (Table 2).

The results revealed that weather condition variables were all significant predictors of PM_2.5_ concentrations, which aligns with the existing literature [64,65]. Wind direction, wind speed, temperature, and dew points are positively associated with PM_2.5_ measurements, and wind gust has negative effects on PM_2.5_ concentration. Temperature mainly affects the diffusion and dilution ability of the atmosphere [66]. The response of PM_2.5_ mass concentration to temperature is complex and varies by different PM_2.5_ components [67]. Sulfate concentrations are expected to increase with increasing temperature because of faster SO2 oxidation [68], which constitute the main fractions of PM_2.5_ mass concentration in the Dallas area [69]. Non-volatile secondary particulate matter also generally increases at higher temperatures due to increased gas-phase reaction rates [68]. Air humidity is likely to hold a nonlinear relationship with the air pollution level. When the humidity is low, it is conducive to the adhesion of atmospheric particulate matter on water vapor, thus leading to the accumulation of air pollutants [66]. Nonetheless, once air humidity rises to form the effective precipitation, the wet deposition processes will remove air pollutants by rainfall. Over the sampling period, the overall humidity condition was dry. Thus, PM_2.5_ concentrations tend to be higher with rising humidity. Wind speed determines the migration speed of pollutants. Low wind speed and strong subsidence have been proven to favor PM_2.5_ accumulation [70]. In addition, most of the wind direction came from the southeast, which facilitates the blowing of the pollution from South Dallas, where heavy traffic and clusters of the industry are located, to Denton. Extreme wind speed, as represented by the wind gust, is the atmospheric dynamic factor that can cause more atmospheric motion and turbulence, and thus improve the diffusion and dilution of air pollutant.

Among all the traffic-related variables, only the distance to major roads (interstate highway) is significant, whereas the distance to minor roads (including avenues and streets) and distance to bus stops were not profound. I-35 is dominated by heavy-duty vehicles (predominantly diesel-powered), while local avenues and streets are mainly used by light-duty vehicles for the daily commute. Although diesel vehicles compose only a small fraction of the mobile fleet, its PM mass emissions are 1–2 orders of magnitude higher than gasoline engines [71,72]. A study that collected PM_2.5_ measures from two relatively close stations reported that the pollution emitted from heavy-duty vehicles mainly determined the PM_2.5_ concentration, whereas contributions from light-duty vehicles were negligible due to the imbalanced emission in black carbon [73]. This is in line with our observation that the distance to major roads is a more important factor than the other two variables. Another noticeable fact is the newly built Buc-ee’s—a supersized highway pit stop with 80–120 fueling positions—is about 3 miles away from the UNT campus. Many diesel vehicle drivers use Buc-ee’s as their stop site, which results in frequent acceleration, deceleration, and idling: the main factors in aggravating vehicle emissions [74]. 

All urban morphology-related variables that have been calculated under the new wind wedge system are significant. Building height was in a positive relationship with PM_2.5_ measurements. Building footprint, implying the development density, also showed a positive but weaker correlation. Thus, the greater the surface area of buildings and the higher the building height cause more PM_2.5_ accumulations, which is partly due to emissions created by increased activity in the surrounding area. In addition, the significant relationships imply the presence of an urban canyon effect in this suburban area, whereby generally poor airflow due to higher density and the height of buildings can cause pollutants to be trapped at the street level. Furthermore, the trapped effect can be intensified by the increasing depth of the street canyons. Previous research also suggests that the street canyon phenomenon may not only be observed in much more highly developed cities, such as Hong Kong [75] and central London [76] but can exist in urban areas where the extension is vast but with most of the buildings less than three stories tall [77]. Finally, large coverage of an impervious surface has been a well-documented influence on the urban heat island effect [78]. The heat island-induced higher temperature could intensify the capping effect of temperature inversions, which elevates the pollution level [79].

Vegetation footprint was negatively related to PM_2.5_ concentrations, indicating that areas with more green spaces tend to have improved air quality conditions. Urban trees are important short-and long-term landscape sinks for elemental carbon [80]. They also mitigate the urban heat island effect by the function of evapotranspiration and reduce temperature inversion-induced pollution. However, vegetation height was in the inverse relationship with the PM_2.5_ level, implying that high-level trees have an adverse effect on air quality. A recent article concluded that the net effects of vegetation vary by each built-up environment through a literature review on neighborhood air quality modifications by green interventions [81]. It suggests that high-level trees being placed along the two sides of the street can lead to air quality deterioration in a street canyon. On the UNT campus, the average tree canopy is above 3 m based on LiDAR detection. A distinct avenue style of planting can be observed from the aerial image-based classification. High-level trees mingled with buildings in a street canyon may act as obstacles to wind flow between the air above the building roof and within the canyon and hence result in accumulated pollutants inside the street canyon [76,81]. However, outside the Denton city core area, where the built environment is featured by sparsely distributed buildings or traffic corridors that are open with generally detached manmade structures, vegetations act as the green barrier that can result in a direct reduction of pollutant concentrations [82,83]. This explains why the larger footprint of green space can mitigate air pollution.

Our final consideration was to compare how the urban morphology variables act differently to the response variable (i.e., PM_2.5_) when they are calculated using different systems: one using the wind wedge system and one using the traditional circular buffers. All variables calculated using the circular buffers show a lower significance level as compared to the ones with wind wedge. Building height was not evaluated as statistically significant. This implies that how urban morphology influences air pollution concentration partly depends on simultaneous wind behavior. This is in line with findings from previous studies: as the wind changes continuously, the retention or flow of the air pollutants changes drastically as well [84]. The complex interplay among wind, urban 3D morphology, and other natural and human environment elements should be inclusively incorporated to account for air pollution dispersal.

## 5. Conclusions

As compared to the typical fixed-site modeling approach, mobile monitoring is superior in its spatial density of measurements. The number of concentration estimates for modeling is 3673 over the 10-day sampling. Our approach is also notably different in how we assembled explanatory variables. First, we included the wind wedge system, making it possible to investigate the effects of wind direction and speed on variable development. Second, our study area is the campus size that makes it possible to quantify the landscape in the vertical dimension than the typical horizontal plane. The LiDAR-derived building height and vegetation height give better information on how 3D urban structure affects pollutant dispersion.

One limitation of mobile monitoring over fixed-site monitoring is its inability to conduct long-term observation. The choice of measurement time, although carefully chosen to capture the variation in traffic flow (the major emission source), may have missed potential temporal hotspots. Another related limitation is the need of high number of repeated measures to access the temporal and spatial variability of air pollutants [85]. However, there is no standard on how many runs should be conducted to accurately represent the background concentration, as the desired number of runs vary by the pollution level [86]. Based on this first survey data, it is possible to conduct a representativeness analysis to provide guidance for future studies. A further limitation is a mismatch in the timestamp between certain explanatory variables and the measurement time. Lacking a sufficient temporal characterization of explanatory variables can be problematic and make identified correlations somewhat questionable. For example, the “distance to minor roads” variable may have benefited from the inclusion of real-time traffic flow, and the “distance to bus stops” variable could be more valuable if the bus schedule is incorporated. This also signifies that the dilemma of mobile monitoring is no longer the acquisition of fine-resolution, high-frequency monitoring data, but rather the absence of suitable environmental parameters with matching spatio-temporal scales. Thus, mitigating these limitations would require collaborative efforts among various data collection and production agencies.

## Figures and Tables

**Table 1:** List of the explanatory variables and their descriptive statistics.

Variables	Acronym	Unit	Data Range
**Meteorological Condition**			
Wind direction	WDIR	degrees (°)	163.3 ± 101.9
Wind speed	WSP	kilometer per hour	15.6 ± 10.1
Wind gust	GST	kilometer per hour	6.4 ± 16.6
Temperature	T	Celsius degree (°C)	9.2 ± −9.6
Dew point	H	Celsius degree (°C)	3.8 ± −8.5
**Proximity to Emission Sources**			
Distance to major roads	Dis_major_	meter	664.8 ± 269.2
Distance to minor roads	Dis_minor_	meter	8.1 ± 22.1
Distance to bus stops	Dis_bus_	meter	67.2 ± 55.2
**Urban Morphology**			
**Wind Wedge**			
Vegetation footprint	VegFp_wedge_	square meter	1,190,152.0 ± 753,784.7
Building footprint	BuildFp_wedge_	square meter	447,183.3 ± 264,025.7
Vegetation height	VegHt_wedge_	meter	0.9 ± 0.4
Building height	BuildHt_wedge_	meter	0.5 ± 0.4
**Circular Buffer**			
Vegetation footprint	VegFp_buffer_	square meter	14,845,468.9 ± 10,916,456.3
Building footprint	BuildFp_buffer_	square meter	5,880,245.9 ± 3,449,631.6
Vegetation height	VegHt_buffer_	meter	0.9 ± 0.2
Building height	BuildHt_buffer_	meter	0.6 ± 0.4

**Table 2:** Results of fixed effects panel model.

Variables	Coefficient Estimate	Standard Error	*p* Value
**Meteorology**			
Wind direction	1.25	0.54	*
Wind speed	12.90	1.54	***
Wind gust	−17.47	0.96	***
Temperature	4.79	0.68	***
Dew point	6.25	0.69	***
**Proximity to Emission Sources**			
Distance to major roads	−0.17	0.29	***
Distance to minor roads	3.08	0.34	
Distance to bus stops	0.03	0.29	
**Urban Morphology**			
**Wind wedge**			
Vegetation footprint	−6.64	0.80	***
Building footprint	1.21	0.61	*
Vegetation height	6.29	0.52	***
Building height	11.38	0.93	***
**Circular buffer**			
Vegetation footprint	90.58	6.27	*
Building footprint	94.87	6.21	*
Vegetation height	−1.86	0.79	*
Building height	1.61	1.55	

Note: * denotes significance level of 0.05; ** denotes significance level of 0.01; and *** denotes significance level of 0.001.

## Supplementary Materials

The following are available online at https://www.mdpi.com/1660-4601/17/14/4914/s1, File S1: The raw data of mobile monitoring surveys, File S2: The aggregated data of mobile monitoring surveys, File S3: Methodology and results of land cover and land use classification.

## References

[1] Gotschi T., Heinrich J., Sunyer J., Künzli N. (2008). Long-Term Effects of Ambient Air Pollution on Lung Function. Epidemiology.

[2] Health Effects Institute (2018). State of Global Air 2018.

[3] Huang J., Pan X., Guo X., Li G. (2018). Impacts of air pollution wave on years of life lost: A crucial way to communicate the health risks of air pollution to the public. Environ. Int..

[4] UN DESA (2015). World Urbanization Prospects: The 2014 Revision.

[5] World Health Organization (2016). Global Urban Ambient Air Pollution Database (Update 2016).

[6] Dionisio K.L., Rooney M.S., Arku R.E., Friedman A.B., Hughes A.F., Vallarino J., Agyei-Mensah S., Spengler J.D., Ezzati M. (2010). Within-Neighborhood Patterns and Sources of Particle Pollution: Mobile Monitoring and Geographic Information System Analysis in Four Communities in Accra, Ghana. Environ. Health Perspect..

[7] Van Vliet E.D.S., Kinney P.L. (2007). Impacts of roadway emissions on urban particulate matter concentrations in sub-Saharan Africa: New evidence from Nairobi, Kenya. Environ. Res. Lett..

[8] Hu X., Waller L.A., Al-Hamdan M.Z., Crosson W.L., Estes M.G., Estes S.M., Quattrochi D.A., Sarnat J.A., Liu Y. (2013). Estimating ground-level PM2.5 concentrations in the southeastern U.S. using geographically weighted regression. Environ. Res..

[9] Pickett S.T., Cadenasso M.L., Grove J.M., Nilon C.H., Pouyat R., Zipperer W.C., Costanza R. (2001). Urban Ecological Systems: Linking Terrestrial Ecological, Physical, and Socioeconomic Components of Metropolitan Areas. Annu. Rev. Ecol. Syst..

[10] Peters A., Von Klot S., Heier M., Trentinaglia I., Hörmann A., Wichmann H.E., Lowel H. (2004). Exposure to Traffic and the Onset of Myocardial Infarction. N. Engl. J. Med..

[11] Apte J., Messier K.P., Gani S., Brauer M., Kirchstetter T.W., Lunden M.M., Marshall J.D., Portier C.J., Vermeulen R.C., Hamburg S.P. (2017). High-Resolution Air Pollution Mapping with Google Street View Cars: Exploiting Big Data. Environ. Sci. Technol..

[12] Jerrett M., Arain A., Kanaroglou P., Beckerman B., Potoglou D., Sahsuvaroglu T., Morrison J., Giovis C., Arain M.A. (2004). A review and evaluation of intraurban air pollution exposure models. J. Expo. Sci. Environ. Epidemiol..

[13] Landrigan P.J., Fuller R., Acosta N.J., Adeyi O., Arnold R., Basu N., Baldé A.B., Bertollini R., Bose-O’Reilly S., Boufford J.I. (2018). The Lancet Commission on pollution and health. Lancet.

[14] Nieuwenhuijsen M. (2016). Urban and transport planning, environmental exposures and health-new concepts, methods and tools to improve health in cities. Environ. Health.

[15] Padhi B.K., Padhy P.K. (2008). Assessment of Intra-urban Variability in Outdoor Air Quality and its Health Risks. Inhal. Toxicol..

[16] Ramanathan V., Feng Y. (2009). Air pollution, greenhouse gases and climate change: Global and regional perspectives. Atmos. Environ..

[17] Saksena S., Singh P., Prasad R.K., Prasad R., Malhotra P., Joshi V., Patil R. (2003). Exposure of infants to outdoor and indoor air pollution in low-income urban areas—A case study of Delhi. J. Expo. Sci. Environ. Epidemiol..

[18] Wheeler A.J., Smith-Doiron M., Xu X., Gilbert N.L., Brook J.R. (2008). Intra-urban variability of air pollution in Windsor, Ontario—Measurement and modeling for human exposure assessment. Environ. Res..

[19] Dimakopoulou K., Gryparis A., Katsouyanni K. (2017). Using spatio-temporal land use regression models to address spatial variation in air pollution concentrations in time series studies. Air Qual. Atmos. Health.

[20] Williams R., Kilaru V., Conner T., Clements A., Colon M., Breen M., Bash J., Duvall R., Szykman J., Landis M. (2018). New Paradigm for Air Pollution Monitoring: Emerging Sensor Technologies 2014–2018 Progress Report.

[21] Hoek G., Beelen R., De Hoogh K., Vienneau D., Gulliver J., Fischer P., Briggs D. (2008). A review of land-use regression models to assess spatial variation of outdoor air pollution. Atmos. Environ..

[22] Hankey S., Marshall J.D. (2015). Land Use Regression Models of On-Road Particulate Air Pollution (Particle Number, Black Carbon, PM2.5, Particle Size) Using Mobile Monitoring. Environ. Sci. Technol..

[23] Tang R., Blangiardo M., Gulliver J. (2013). Using Building Heights and Street Configuration to Enhance Intraurban PM10, NOX, and NO2Land Use Regression Models. Environ. Sci. Technol..

[24] Li X., Liu W., Chen Z., Zeng G., Hu C., León T., Liang J., Huang G., Gao Z., Li Z. (2015). The application of semicircular-buffer-based land use regression models incorporating wind direction in predicting quarterly NO 2 and PM 10 concentrations. Atmos. Environ..

[25] Naughton O., Donnelly A., Nolan P., Pilla F., Misstear B., Broderick B. (2018). A land use regression model for explaining spatial variation in air pollution levels using a wind sector based approach. Sci. Total. Environ..

[26] Ghassoun Y., Löwner M.-O. (2017). Land use regression models for total particle number concentrations using 2D, 3D and semantic parameters. Atmos. Environ..

[27] U.S. Census Bureau Population and Housing Unit Estimates Tables. https://www.census.gov/programs-surveys/popest/data/tables.html.

[28] UNT 2018 Fact Book. https://institutionalresearch.unt.edu/fact-book/enrollment.

[29] North Texas Daily. https://www.ntdaily.com/texas-cuts-to-air-quality-planning-risk-increasing-pollution-in-denton/.

[30] Sales J. (2007). Determining the Suitability of Functional Landscapes and Wildlife Corridors Utilizing Conservation GIS Methods in Denton County, Texas. Master’s Thesis.

[31] An R., Yu H. (2018). Impact of ambient fine particulate matter air pollution on health behaviors: A longitudinal study of university students in Beijing, China. Public Health.

[32] Rajper S.A., Ullah S., Wang J. (2018). Exposure to air pollution and self-reported effects on Chinese students: A case study of 13 megacities. PLoS ONE.

[33] Hien P.D., Bac V.T., Tham H.C., Nhan D.D., Vinh L.D. (2002). Influence of meteorological conditions on PM2.5 and PM2.5–10 concentrations during the monsoon season in Hanoi, Vietnam. Atmos. Environ..

[34] Xu Y., Xue W., Lei Y., Zhao Y., Cheng S., Ren Z., Huang Q. (2018). Impact of Meteorological Conditions on PM2.5 Pollution in China during Winter. Atmosphere.

[35] He J., Gong S., Yu Y., Yu L., Wu L., Mao H., Song C., Zhao S., Liu H., Li X. (2017). Air pollution characteristics and their relation to meteorological conditions during 2014–2015 in major Chinese cities. Environ. Pollut..

[36] Wang J., Ogawa S. (2015). Effects of Meteorological Conditions on PM2.5 Concentrations in Nagasaki, Japan. Int. J. Environ. Res. Public Health.

[37] Hernandez G., Berry T.A., Wallis S., Poyner D., Juan L. (2017). Temperature and humidity effects on particulate matter concentrations in a sub-tropical climate during winter. Proceedings of the International Conference of the Environment, Chemistry and Biology (ICECB 2017).

[38] Zhang C., Ni Z., Ni L. (2015). Multifractal detrended cross-correlation analysis between PM2.5 and meteorological factors. Phys. A Stat. Mech. Its Appl..

[39] Barmpadimos I., Hueglin C., Keller J., Henne S., Prevot A.S.H. (2011). Influence of meteorology on PM10 trends and variability in Switzerland from 1991 to 2008. Atmos. Chem. Phys. Discuss..

[40] Shi P., Zhang G., Kong F., Chen D., Azorin-Molina C., Guijarro J. (2019). Variability of winter haze over the Beijing-Tianjin-Hebei region tied to wind speed in the lower troposphere and particulate sources. Atmos. Res..

[41] Xie J., Liao Z., Fang X., Xu X., Wang Y., Zhang Y., Liu J., Fan S., Wang B. (2019). The characteristics of hourly wind field and its impacts on air quality in the Pearl River Delta region during 2013–2017. Atmos. Res..

[42] Yassin M.F. (2012). Numerical modeling on air quality in an urban environment with changes of the aspect ratio and wind direction. Environ. Sci. Pollut. Res..

[43] Pushpawela B., Jayaratne R., Morawska L. (2019). The influence of wind speed on new particle formation events in an urban environment. Atmos. Res..

[44] Arain M.A., Blair R., Finkelstein N., Brook J., Sahsuvaroglu T., Beckerman B., Zhang L., Jerrett M. (2007). The use of wind fields in a land use regression model to predict air pollution concentrations for health exposure studies. Atmos. Environ..

[45] Shi Y., Lau K.K.-L., Ng E. (2017). Incorporating wind availability into land use regression modelling of air quality in mountainous high-density urban environment. Environ. Res..

[46] Lowicki D. (2019). Landscape pattern as an indicator of urban air pollution of particulate matter in Poland. Ecol. Indic..

[47] NOAA. https://www.ncdc.noaa.gov/isd/data-access.

[48] Askariyeh M.H., Zietsman J., Autenrieth R. (2019). Traffic contribution to PM2.5 increment in the near-road environment. Atmos. Environ..

[49] Karner A., Eisinger D.S., Niemeier D.A. (2010). Near-Roadway Air Quality: Synthesizing the Findings from Real-World Data. Environ. Sci. Technol..

[50] Dewinter J.L., Brown S.G., Seagram A.F., Landsberg K., Eisinger D.S. (2018). A national-scale review of air pollutant concentrations measured in the U.S. near-road monitoring network during 2014 and 2015. Atmos. Environ..

[51] Keuken M., Moerman M., Voogt M., Blom M., Weijers E., Röckmann T., Dusek U. (2013). Source contributions to PM2.5 and PM10 at an urban background and a street location. Atmos. Environ..

[52] Rouse J.W., Haas R.H., Schell J.A., Deering D.W. Monitoring vegetation systems in the Great Plains with ERTS. Proceedings of the Third earth resources technology satellite-1 symposium.

[53] Wu J., Xie W., Li W., Li J. (2015). Effects of Urban Landscape Pattern on PM2.5 Pollution—A Beijing Case Study. PLoS ONE.

[54] Zupancic T., Westmacott C., Bulthuis M. (2015). The Impact of Green Space on Heat and Air Pollution in Urban Communities: A Meta-Narrative Systematic Review.

[55] Weng Q., Yang S. (2006). Urban Air Pollution Patterns, Land Use, and Thermal Landscape: An Examination of the Linkage Using GIS. Environ. Monit. Assess..

[56] Schlegel M. (1987). 4.5 National Weather Services. Thermodynamical and Dynamical Structures of the Global Atmosphere.

[57] Ahn S.C., Schmidt P. (1995). Efficient estimation of models for dynamic panel data. J. Econ..

[58] Hausman J.A. (1978). Specification Tests in Econometrics. Econom. J. Econom. Soc..

[59] Greene W.H. (1990). Econometric Analysis.

[60] Wang Y., Chen J., Wang Q., Qin Q., Ye J., Han Y., Li L., Zhen W., Zhi Q., Zhang Y. (2019). Increased secondary aerosol contribution and possible processing on polluted winter days in China. Environ. Int..

[61] Zhou S., Lin R. (2019). Spatial-temporal heterogeneity of air pollution: The relationship between built environment and on-road PM2.5 at micro scale. Transp. Res. Part D Transp. Environ..

[62] Hasheminassab S., Pakbin P., Delfino R.J., Schauer J.J., Sioutas C. (2014). Diurnal and seasonal trends in the apparent density of ambient fine and coarse particles in Los Angeles. Environ. Pollut..

[63] Kimbrough S., Baldauf R.W., Hagler G., Shores R.C., Mitchell W., Whitaker D.A., Croghan C.W., Vallero D.A., Kimbrough S. (2012). Long-term continuous measurement of near-road air pollution in Las Vegas: Seasonal variability in traffic emissions impact on local air quality. Air Qual. Atmos. Health.

[64] Chaloulakou A., Kassomenos P., Spyrellis N., Demokritou P., Koutrakis P. (2003). Measurements of PM10 and PM2.5 particle concentrations in Athens, Greece. Atmos. Environ..

[65] Zhang H., Wang Y., Park T.W., Deng Y. (2017). Quantifying the relationship between extreme air pollution events and extreme weather events. Atmos. Res..

[66] Zhang Y. (2019). Dynamic effect analysis of meteorological conditions on air pollution: A case study from Beijing. Sci. Total. Environ..

[67] Tai A.P.K., Mickley L.J., Jacob D.J. (2010). Correlations between fine particulate matter (PM2.5) and meteorological variables in the United States: Implications for the sensitivity of PM2.5 to climate change. Atmos. Environ..

[68] Aw J., Kleeman M.J. (2003). Evaluating the first-order effect of intraannual temperature variability on urban air pollution. J. Geophys. Res. Space Phys..

[69] Hand J.L., Schichtel B.A., Pitchford M., Malm W.C., Frank N.H. (2012). Seasonal composition of remote and urban fine particulate matter in the United States. J. Geophys. Res. Space Phys..

[70] Hsu C.-H., Cheng F.-Y. (2016). Classification of weather patterns to study the influence of meteorological characteristics on PM2.5 concentrations in Yunlin County, Taiwan. Atmos. Environ..

[71] Kittelson D.B. (1998). Engines and nanoparticles. J. Aerosol Sci..

[72] Shields L.G., Suess D.T., Prather K.A., Prather K. (2007). Determination of single particle mass spectral signatures from heavy-duty diesel vehicle emissions for PM2.5 source apportionment. Atmos. Environ..

[73] Sofowote U., Healy R., Su Y., Debosz J., Noble M., Munoz A., Jeong C.-H., Wang J., Hilker N., Evans G. (2018). Understanding the PM2.5 imbalance between a far and near-road location: Results of high temporal frequency source apportionment and parameterization of black carbon. Atmos. Environ..

[74] Chen C., Huang C., Jing Q., Wang H., Pan H., Li L., Zhao J., Dai Y., Huang H., Schipper L. (2007). On-road emission characteristics of heavy-duty diesel vehicles in Shanghai. Atmos. Environ..

[75] Chan L., Kwok W., Lee S.-C., Chan C. (2001). Spatial variation of mass concentration of roadside suspended particulate matter in metropolitan Hong Kong. Atmos. Environ..

[76] Jeanjean A.P., Buccolieri R., Eddy J., Monks P., Leigh R.J. (2017). Air quality affected by trees in real street canyons: The case of Marylebone neighbourhood in central London. Urban For. Urban Green..

[77] Wang C., Li Q., Wang Z. The Residence time of pollutants emitted within the urban canopy influenced by street canyon geometry and emission conditions. Proceedings of the 100th American Meteorological Society Annual Meeting.

[78] Chen X., Zhao H.-M., Li P.-X., Yin Z.-Y. (2006). Remote sensing image-based analysis of the relationship between urban heat island and land use/cover changes. Remote. Sens. Environ..

[79] Gramsch E., Caceres D., Oyola P., Reyes F., Vasquez Y., Rubio M., Sánchez G. (2014). Influence of surface and subsidence thermal inversion on PM2.5 and black carbon concentration. Atmos. Environ..

[80] Rindy J.E., Ponette-González A.G., Barrett T.E., Sheesley R.J., Weathers K.C. (2019). Urban Trees Are Sinks for Soot: Elemental Carbon Accumulation by Two Widespread Oak Species. Environ. Sci. Technol..

[81] Abhijith K., Kumar P., Gallagher J., McNabola A., Baldauf R., Pilla F., Broderick B., Di Sabatino S., Pulvirenti B. (2017). Air pollution abatement performances of green infrastructure in open road and built-up street canyon environments—A review. Atmos. Environ..

[82] De Carvalho R.M., Szlafsztein C.F. (2019). Urban vegetation loss and ecosystem services: The influence on climate regulation and noise and air pollution. Environ. Pollut..

[83] Van Ryswyk K., Prince N., Ahmed M., Brisson E., Miller J.D., Villeneuve P.J. (2019). Does urban vegetation reduce temperature and air pollution concentrations? Findings from an environmental monitoring study of the Central Experimental Farm in Ottawa, Canada. Atmos. Environ..

[84] Yang J., Shi B., Shi Y., Marvin S., Zheng Y., Xia G. (2020). Air pollution dispersal in high density urban areas: Research on the triadic relation of wind, air pollution, and urban form. Sustain. Cities Soc..

[85] Gozzi F., Della Ventura G., Marcelli A. (2016). Mobile monitoring of particulate matter: State of art and perspectives. Atmos. Pollut. Res..

[86] Van Poppel M., Peters J., Bleux N. (2013). Methodology for setup and data processing of mobile air quality measurements to assess the spatial variability of concentrations in urban environments. Environ. Pollut..

