# Supplementary material for: Monitoring, Mapping, and Modeling Spatial–Temporal Patterns of PM2.5 for Improved Understanding of Air Pollution Dynamics Using Portable Sensing Technologies
Source: Int J Environ Res Public Health. 2020 Jul 8;17(14):4914. doi: 10.3390/ijerph17144914 (PMC7400490; doi:10.3390/ijerph17144914)
Supplement: Supplementary file 1 [file ijerph-17-04914-s001.zip › Supplementary File 3.docx]

**Supplementary File S1: Classification method and results of NAIP image**

Eight land cover and land use (LULC) types encompassing the study area, separately barren, grassland, high-intensity urban, low-intensity urban, major roads, minor roads, forest, and water, were included in the classification. This classification was modified from the Anderson Land Cover Classification System and followed the classification system used by Multi-Resolution Land Characteristics Consortium National Land Cover Database (acquired from the USDA Geospatial Data Gateway; MRLC 2014).

1. High-intensity urban: a highly developed area where impervious surfaces account for 80% to 100% of the total cover, mostly containing commercial and industrial property that appears to have a higher reflectance value than the low-intensity urban (MRLC 2014);
2. Low-intensity urban: a less developed area where impervious surfaces account for 50% to 80% of the total cover, mostly containing residential areas (Homer et al., 2007).
3. Roads: roads were not included in the urban classes due to their spectral signatures being similar to bare soils. The major roads primarily comprise highways, and minor roads are local paved roads with one or two lanes, dirt and gravel roads.
4. Grassland: open areas of crops planted by farmers, ranches, or other short vegetative growth in fields and bare patches of lands that lack intense vegetation.
5. Bare lands: areas that have not been used for agriculture or pasture for animals, and are usually land stocks for urban expansion;
6. Forest: an area that is dominated mostly by tree cover.
7. Water: includes lakes, ponds, rivers, streams, and creeks that are visually identifiable on the NAIP imagery.

We included the Normalized Difference Vegetation Index (NDVI) in classification. It is a quantitative index of greenness ranging from 0-1 where 0 represents minimal or no greenness and 1 represents maximum greenness. It is added as the fifth band to the NAIP imagery.

For training samples, we randomly placed 240 samples by following the rule that training samples should be representative of the land-cover types in the study area. More than 30 samples were collected for each class, with each sample greater than 1.5x1.5 pixels in size and homogeneous in nature. We further visually interpreted their LULC types based on NAIP imagery. The labels of those samples were cross-checked by an independent analyst who received the same training to ensure the quality.

Classifications were performed with the Maximum Likelihood Classifier (MLC) using the combined four raw spectral bands and one spectral indices. The MLC is a parametric method widely used as a reference for its computational simplicity and robustness. All classes were assigned to have equal prior probability and no pixel would be rejected for a class assignment.

For accuracy assessments, a hundred of validation samples were randomly place over the study area and visually interpreted by following the same procedure as the training samples. A confusion matrix and associated accuracy measures were generated, including the overall accuracy, kappa, producer’s accuracy, and user’s accuracy (Table S1).

**Table S1:** Confusion matrix for MLC classification.

|  | **Actual Category: Ground Truth** | | | | | | |  |
| --- | --- | --- | --- | --- | --- | --- | --- | --- |
| **Classified Category** | High Intensity Urban | Low Intensity Urban | Grassland | Barren | Forest | Water | Total | User's Accuracy |
| High Intensity Urban | 7 | 0 | 0 | 0 | 0 | 0 | 7 | 100% |
| Low Intensity Urban | 4 | 4 | 0 | 1 | 0 | 0 | 9 | 44% |
| Grassland | 0 | 6 | 40 | 6 | 2 | 0 | 54 | 74% |
| Barren | 0 | 0 | 0 | 3 | 0 | 0 | 3 | 100% |
| Forest | 0 | 2 | 4 | 0 | 21 | 0 | 27 | 78% |
| Water | 0 | 0 | 0 | 0 | 0 | 0 | 0 | NAN |
| Total | 11 | 12 | 44 | 10 | 23 | 0 | 100 |  |
| Producer's Accuracy | 64% | 33% | 91% | 30% | 91% | NAN |  |  |

In subsequent analysis, the footprint of buildings was represented by the high-intensity and low-intensity urban. Forest and grassland were used to derive the green space distribution (Figure S1).


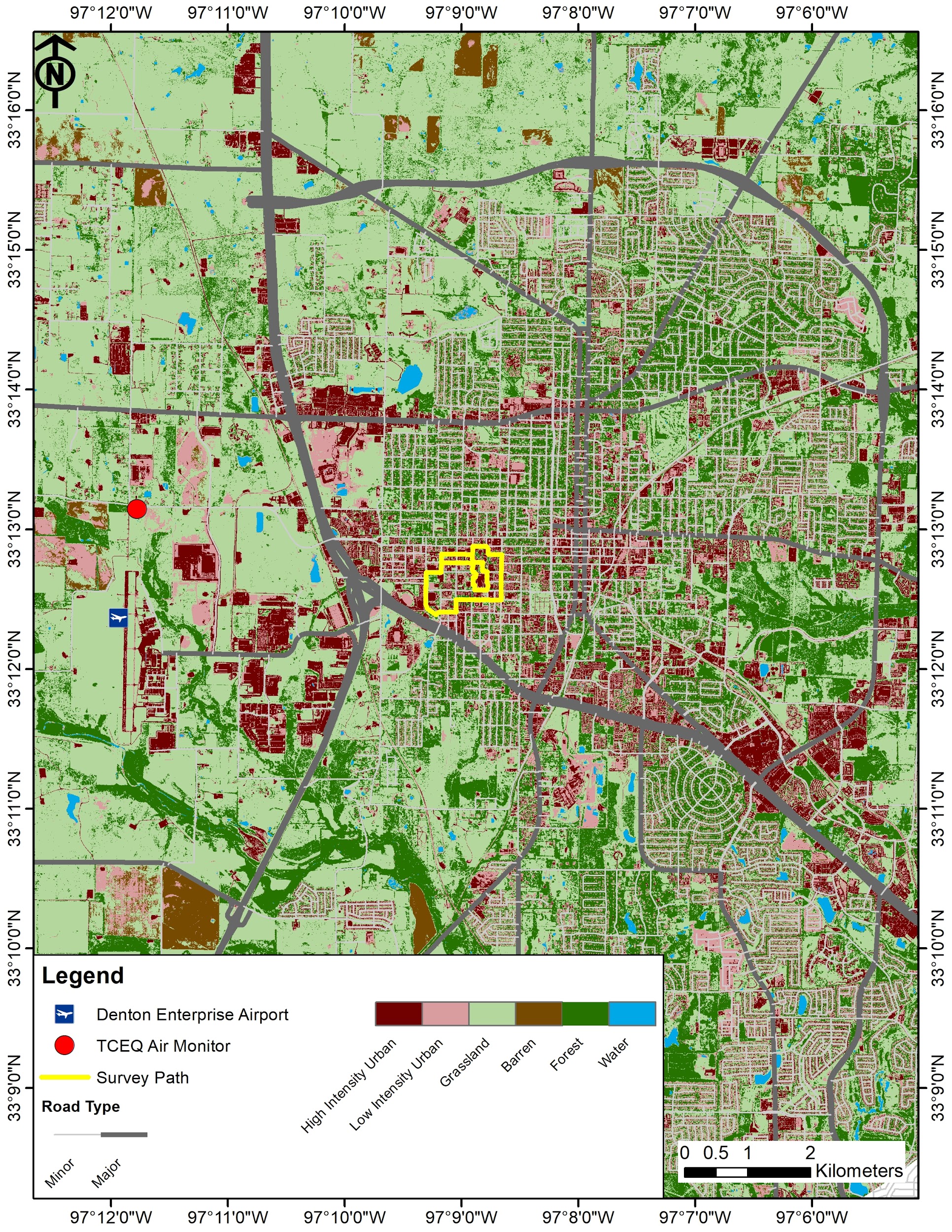


**Figure S1:** Map of land cover type on UNT campus and the surrounding areas.

**References:**

Homer, C.; Dewitz, J.; Fry, J.; Wickham, J.D. Completion of the 2001 National LandCover Database for the Conterminous United States. *Photogramm. Eng. Remote Sens.* **2007**, *73*, 337–341.

MRLC 2014. Multi-Resolution Land Characteristics National Land Cover Database. Available online: [**http://www.mrlc.gov/index.php**](http://www.mrlc.gov/index.php) (accessed on 26 November 2019).

USDA Geospatial Data Gateway. Available online: https://gdg.sc.egov.usda.gov/ (accessed on 26 November 2019).
